# Determinants analysis of outpatient service utilisation in Georgia: can the approach help inform benefit package design?

**DOI:** 10.1186/s12961-017-0197-5

**Published:** 2017-05-02

**Authors:** George Gotsadze, Wenze Tang, Natia Shengelia, Akaki Zoidze

**Affiliations:** 1 Curatio International Foundation, 37d Chavchavadze Ave., 0162 Tbilisi, Georgia; 2 Semel Institute for Neuroscience and Human Behavior, 760 Westwood Plaza, CA Los Angeles, 90024 United States of America

**Keywords:** Non-communicable diseases, Outpatient service utilisation, Behavioural model, Benefits package, Determinants of service use

## Abstract

**Background:**

The healthcare financing reforms initiated by the Government of Georgia in 2007 have positively affected inpatient service utilisation and enhanced financial protection, especially for the poor, but they have failed to facilitate outpatient service use among chronic patients. Non-communicable diseases significantly affect Georgia’s ageing population. Consequently, in this paper, we look at the evidence emerging from determinants analysis of outpatient service utilisation and if the finding can help identify possible policy choices in Georgia, especially regarding benefit package design for individuals with chronic conditions.

**Methods:**

We used Andersen’s behavioural model of health service utilisation to identify the critical determinants that affect outpatient service use. A multinomial logistic regression was carried out with complex survey design using the data from two nationally representative cross-sectional population-based health utilisation and expenditure surveys conducted in Georgia in 2007 and 2010, which allowed us to assess the relationship between the determinants and outpatient service use.

**Results:**

The study revealed the determinants that significantly impede outpatient service use. Low income, 45- to 64-year-old Georgian males with low educational attainment and suffering from a chronic health problem have the lowest odds for service use compared to the rest of the population.

**Conclusions:**

Using Andersen’s behavioural model and assessing the determinants of outpatient service use has the potential to inform possible policy responses, especially those driving services use among chronic patients. The possible policy responses include reducing financial access barriers with the help of public subsidies for sub-groups of the population with the lowest access to care; focusing/expanding state-funded benefits for the most prevalent chronic conditions, which are responsible for the greatest disease burden; or supporting chronic disease management programs for the most prevalent chronic diseases and for special age groups aimed at the timely detection, education and management of chronic patients.

## Background

The healthcare financing reforms initiated by the Government of Georgia in 2007 (which are described in detail later in the paper) had a positive impact on reducing expenditure on inpatient services and total household healthcare costs, thus increasing the probability of receiving free outpatient benefits for program beneficiaries [1]. However, on an outpatient level, the program improved utilisation and reduced costs only for patients with acute health needs, with chronic patients only marginally benefiting from these reforms, and even then only those who faced an exacerbation of their illnesses in the 30 days that preceded the survey [2]. These findings suggest that the reforms did not adequately address the needs of the population where chronic diseases are prevalent. The above results therefore highlight the need to further explore this phenomenon by perfoming a thorough analysis of the determinants of outpatient service use, especially among individuals suffering from chronic health problems.

The challenges posed by non-communicable diseases (NCDs) are well documented as these are responsible for more than 36 million deaths annually, with nearly 80% (29 million) of these deaths occurring in low- and middle-income countries (LMICs) [3]. Ageing populations and increasing life expectancy contribute to an epidemiological transition globally, and chronic conditions are becoming an even bigger challenge especially for LMICs [4]. NCDs also impose significant costs on households, emerging as financial access barriers to care, especially for the poor and disadvantaged. All of this further emphasises the importance of improving financial risk protection against ill health in LMICs and ensuring that NCDs are considered when reforming healthcare financing systems [5]. Therefore, WHO, in its Global Action Plan for the Prevention and Control of NCDs [6], identified the importance of strengthening health systems and addressing the prevention and control of NCDs and the underlying social determinants through people-centred primary healthcare and universal health coverage.

Experience from high-income countries able to control NCDs shows that responses must be comprehensive and multi-sectoral and, among other factors, should also address financial risk protection ensuring equity in access and payments [7]. Considering this, the design of healthcare benefit packages is an important policy tool that can positively affect health services utilisation and ensure socioeconomic equity in service use. Without careful design of benefits, health insurance schemes (national, private, community, etc.) may not assist those who are most in need of financial protection from health service expenses [8].

Consequently, to tackle NCDs, it seems necessary to undertake a careful design of the publicly financed benefit packages, which could be just one policy instrument out of many. For this purpose, we use data from two rounds of Georgia’s population-based and nationally representative health utilisation and expenditure surveys (HUES). Using Andersen’s behavioural model, we try to unpack the determinants impeding outpatient service use among chronic patients. We believe that using Andersen’s behavioural model and unpacking the determinants of service utilisation has the potential to inform the selection of possible policy responses, those focused on chronic patients in Georgia, and making the state-funded benefit package more patient centred. This could facilitate improved access to care in an equitable manner, which could lead to improved and more equitable health outcomes. We also believe that the approaches used in this study may have relevance for other LMICs.

### Country context

Georgia initiated health sector reforms in 1995, soon after its independence from the Soviet Union. The initial reform agenda included changes in healthcare financing and the introduction of a payroll tax along with budget transfers pooled by a newly established single public purchaser. It also separated healthcare financing and provision functions, followed by the autonomisation and eventual privatisation of a provider network. Moreover, it emphasised the importance of public health and established the National Center for Disease Control to lead the public health agenda. However, these initial attempts were not successful in financially protecting Georgian households and securing adequate access to care. Due to limited public financing, out-of-pocket (OOP) payments emerged as a primary source of healthcare financing, which placed a significant financial burden on households and created access barriers to care for many [9].

In 2007, the Government of Georgia embarked on the second phase of its healthcare reforms. Instead of offering a limited package of publicly funded benefits to everyone, the aim of the reforms was to deliver a comprehensive and fully subsidised insurance coverage to the poorest segments of the population (as determined through proxy means testing). For the non-poor population, state-covered services included public health programs (e.g. HIV/AIDS, tuberculosis, immunisation) and narrowly defined benefits on a primary healthcare level with limited diagnostic and/or specialised services. Laboratory and diagnostic tests included full blood count, urinary, blood glucose and creatinine tests, electrocardiogram, and X-rays only for children aged 0–3 years. Specialised services included neurologist, endocrinologist, oncologist, ophthalmologist, otolaryngologist and child orthopaedist consultations [10]. For the non-poor population, state subsidies were also provided for life-threatening medical emergencies, although co-payments from patients amounting to 25–50% were also required [11].

After piloting in two geographic locations during 2007, this new program – Medical Insurance for the Poor (MIP) – was rolled out nationwide and, by the end of 2010, covered almost 20% of the population [12, 13].^1^ The MIP benefit package included (1) urgent outpatient and inpatient treatment inclusive of all necessary diagnostic-laboratory tests to determine the need for hospitalisation; (2) planned in-patient services, with an annual insurance limit of 15,000 GEL (1 GEL ~ US$0.6 US), excluding expenses for cosmetic and aesthetic surgery, resort treatment, sexual disorders, infertility, treatment abroad, sexually transmitted infections, and hepatitis C; (3) chemotherapy and radiation therapy up to a 12,000 GEL annual limit; (4) outpatient visits to specialists with very limited diagnostic and laboratory tests prescribed by a general practitioner on a PHC level; and (5) compensation for delivery costs (up to 400 GEL). Outpatient prescription drug benefits were added to this package from 2010 and included pharmaceuticals from the predefined essential list of medicines, with an annual financial limit of 50 GEL subject to a 50% co-payment by the patient. Consequently, the level of annual public subsidy for outpatient drugs – 25 GEL (approximately US$ 15) – was minimal, especially for chronic patients who spend on average 600 GEL per annum [1, 14, 15]. The benefit coverage under the MIP was delivered by the private insurers through competing contracts with a single public purchaser [1]. As noted above, anyone who does not qualify for MIP is eligible for a ‘basic package’ of services offered throughout Georgia and funded by the single public payer. Thus, the poverty eligibility thresholds became the dividing line between a single public purchaser covering a limited benefit package with significant co-payments for the general population, and multiple private insurers using publicly paid insurance premiums and providing a more comprehensive benefits MIP package with only co-payments on the cost of the predefined list of essential medicines [11].

Several studies evaluated the MIP impact and concluded that the program resulted in improved financial protection for the covered individuals, facilitated access to inpatient and outpatient services for acute patients, and had a positive equity impact by delivering greater financial benefits to the poorest members of society [1, 11, 16]. However, the impact of the MIP on overall outpatient utilisation was minimal (2%) [16]. Moreover, the MIP did not facilitate the use of services among individuals with chronic conditions, which triggered the need to look more carefully at the determinants of service utilisation and uncover the factors that shape the different impact of the MIP on acute and chronic patients seeking outpatient services [2]. Consequently, we decided to focus this paper on exploring these determinants in order to obtain the evidence to inform policy, especially concerning benefit package improvements that could help deliver more patient-centred services, facilitate utilisation growth and improve health outcomes for chronic patients.

## Methods

### Conceptual framework for selection of determinants

Andersen’s behavioural model of health service utilisation, which assumes that the decision to use services is influenced both by an individual and by context-specific factors [17, 18], provides the theoretical framework for our study. In his model, Andersen argues that three groups of factors determine people’s use of health services, including (1) an individual’s predisposition to use services; (2) factors that enable or inhibit use; and (3) an individual’s need for care. The model builds on the analytical process or causal ordering in which predisposing factors include personal characteristics that are not directly related to medical use, but rather influence the likelihood of utilisation. Enabling factors refer to the means that individuals have (or do not have) at their disposal, which could be deployed and used for accessing the services. The need arises from an individual’s health status.

Based on this theory, and after checking variables in our dataset, we decided to test the following determinants in our study. Predisposing factors include an individual’s age, sex, educational attainment, marital status, ethnicity and trust level in a regular source of care facility. Enabling factors include the type and ownership of medical insurance, rurality of a household and its wealth (measured by a family’s monthly consumption level) organised in tercile groups, the type of regular care facility and travel time to reach it and/or the type of closest healthcare facility (if different from the regular care facility), median household age as a proxy characteristic of a household’s age diversity, and district level median OOP cost for outpatient services and physician density per district to account for supply side environmental variables in a given geographic location [19–22]. The patient’s need was operationalised using the variable ‘self-perceived health status in the past 4 weeks prior to the survey’. We added geographic regions to account for environmental differences between regions in the country and, finally, we used disease type and year of survey as confounders. Please refer to Table 1 for a full list of the determinants, their typology and response levels (i.e. individual, household, district and region).

**Table 1:** Descriptive statistics

Level	Variables	Values	%	Mean (SD)	Median	N missing
			*N* = 10,952	*N* = 10,952		
Need						
I	Perceived health status in the past 4 weeks (%)	Very poor/poor	45.53			116
		Fair	40.14			
		Good/very good/excellent	14.34			
Predisposing factors						
I	Age group (%)	<15	8.14			
		15–44	26.82			
		45–64	33.43			
		≥65	31.61			
I	Sex (%)	Male	42.24			
		Female	57.76			
I	Education (%)	College/higher education	18.72			135
		High/technical school	37.54			
		Less than high school	43.74			
I	Marital status (%)	Currently married	56.9			115
		Not married	43.1			
I	Ethnicity (%)	Georgian	89.07			79
		Armenian	4.31			
		Azeri	4.01			
		Others	2.6			
H	Trust in regular source of care facility (%)	Not reported	10.77			
		Little/not at all	2.37			
		Sufficiently	25.22			
		Quite a lot	39.02			
		Very much	22.63			
Enabling factors						
I	Type of insurance (%)	MIP	21.83			148
		Other private insurance	4.19			
		No insurance	73.98			
H	Residence type (%)	Urban	37.87			
		Rural	62.13			
H	Household consumption tercile (%)	High	31.71			
		Medium	34.85			
		Low	33.44			
H	Type of regular source of care facility (%)	Village ambulatory clinic and others	1.69			40
		Polyclinic	7.78			
		Hospital	9.33			
		No regular care	81.2			
H	Travel time to regular source of care facility (minutes)			26.80 (33.70)	20	
H	Median household age			42.72 (17.55)	39	
H	Type of closest facility (%)	Hospital	18.87			97
		Polyclinic	32.6			
		Village ambulatory clinics	47.02			
		Others	1.51			
D	District-level median OOP cost to OP facilities (GEL)			13.50 (10.33)	11.82	8
D	Physician density per district per 1000 population			3.98 (2.54)	3.11	
External environment						
R	Geographic region	Tbilisi	17.26			
		West	41.96			
		East	40.79			
Confounder						
	Year	Year 2010	50.4			
		Year 2007	49.6			
	Disease type	Chronic with exacerbation within 30 days prior to the survey	14.0			
		Chronic illness only	70.3			
		Acute illness only	15.7			

*I*individual level factor, *H* household level factor, *D* district level factor, *R* regional level factor, *MIP* medical insurance for the poor, *GEL* Georgian Lari, *OOP* out-of-pocket, *OP* outpatient

### Data sources

We used a cross-sectional design to analyse the pooled dataset from Georgia’s nationally representative HUES from 2007 and 2010. Both rounds utilised a two-stage stratified random sampling procedure, with census enumeration areas as the primary sampling unit and households as the secondary unit. The sample stratification was based on the classification of settlement types, which took into consideration factors such as urban/rural and cities/towns/villages. A comparable number of households (about 3200) was interviewed in each round, and the response rate was 95% and 89% in 2007 and 2010, respectively. The surveys collected two segments of information, namely (1) general information on both household and individual level; and (2) full health utilisation and expenditure information for each health problem reported. More details about the sampling, survey methodology and questionnaire are available elsewhere [15]. To enrich the analysis with contextual/environmental data, the database was complemented with measures about healthcare inputs (e.g. doctors, nurses, hospital beds, etc.) obtained from official statistical yearbooks [23, 24].

### Study population

**Fig. 1:**
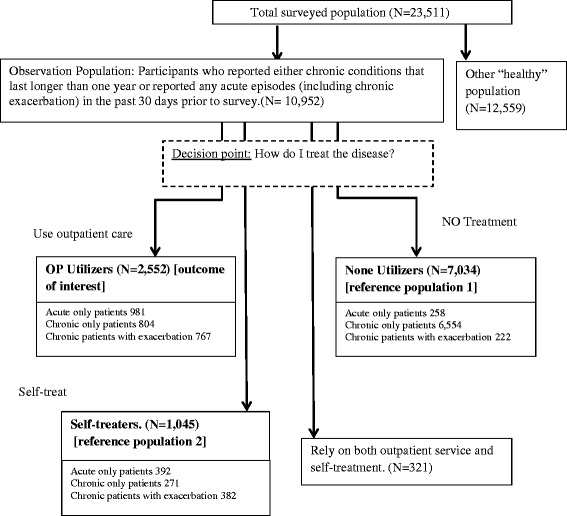
Hypothetical decision tree on outpatient healthcare utilisation

We focused this analysis on survey participants who reported the following diseases: chronic conditions, defined as health problems that lasted longer than 1 year, and acute episodes or exacerbation of chronic diseases occurring during the 30 days prior to the survey. As noted above, previous analysis of utilisation patterns differed dramatically between patients who only reported chronic conditions and patients who only reported acute episodes [2]. Therefore, we controlled for disease type as a significant confounder in the regression (Fig. 1).

### Measures of service utilisation

Our primary outcome of interest concerns outpatient service utilisation (utilisers). However, a significant portion of survey participants relied solely on self-treatment (self-treaters) or did not utilise any outpatient service (non-utilisers). Surveys also captured people who used both self-treatment and outpatient service use (dual users), although the group was too small to allow for robust analysis (Fig. 1). Therefore, to unpack utilisation behaviour along the decision tree, we decided to compare outpatient utilisers against two reference groups, namely ‘non-utilisers’ and ‘self-treaters’.

### Statistical analysis

We first derived descriptive statistics for all of the continuous and categorical variables detailed in Table 1. We then assessed the correlation between continuous variables and made a final decision on the selection and inclusion of variables in the determinants analysis [25]. We did not conduct any bivariate analysis or stepwise analysis because of the limited number of available variables and concern for multiple comparisons [26]. Finally, we used a multinomial multivariate logistic regression with complex survey design [27, 28] to analyse the relationship between the determinants and indicators of outpatient service use. A complex survey design model was explicitly adjusted for differing probabilities of household selection by using primary sampling unit and strata identifiers, which was readily available in the dataset. A syntax file provided in SAS® [29, 30] was used. The statistical significance for the analysis was set at two-sided *P* < 0.05 and a χ^2^ test was used to determine statistical significance in the regression. We used commercially available statistical software SPSS® Version 21.0 for preliminary data preparation and SAS/STAT® Version 9.3 for data analysis.

## Results

A summary of the descriptive statistics for independent variables is presented in Table 1. In the following sections, we discuss the influence of each determinant, as established through the regression analysis and shown in Table 2.

**Table 2:** Determinants of healthcare utilisation among population reporting chronic conditions, acute conditions and chronic conditions with acute exacerbation

			Outpatient utilisers vs. non-utilisers		Outpatient utilisers vs. self-treaters	
Factor	Variable	Level	Odds ratio (95% CL)	*P* value	Odds ratio (95% CL)	*P* value
Need	Perceived health status	Excellent/very good/good	0.64 (0.50–0.82)	0.0004	0.75 (0.56–0.99)	0.0434
		Fair	0.74 (0.64–0.87)	0.0002	0.69 (0.56–0.85)	0.0006
		poor/very poor	1.00		1.00	
Predisposing factors	Age group	0–14	2.02 (1.45–2.81)	<0.001	2.85 (1.85–4.39)	<0.001
		15–44	1.16 (0.95–1.41)	0.1377	1.52 (1.20–1.93)	0.0005
		≥65	1.16 (0.96–1.40)	0.1322	1.27 (0.99–1.63)	0.0590
		45–64	1.00		1.00	
	Education	College or higher	1.27 (1.01–1.60)	0.0447	1.15 (0.89–1.48)	0.2756
		High school/technical school education	1.24 (1.03–1.49)	0.0231	1.17 (0.95–1.44)	0.1388
		Less than high school	1.00		1.00	
	Ethnicity	Azeri	0.95 (0.55–1.62)	0.8391	1.27 (0.43–3.72)	0.6616
		Armenian	1.52 (1.11–2.09)	0.0097	3.60 (1.92–6.74)	0.0001
		Other	0.98 (0.64–1.49)	0.9241	0.63 (0.42–0.96)	0.0332
		Georgian	1.00		1.00	
	Marital status	Married	1.05 (0.90–1.22)	0.5633	0.91 (0.75–1.10)	0.3246
		Others	1.00		1.00	
	Sex	Female	1.15 (1.01–1.30)	0.0351	0.92 (0.78–1.09)	0.3436
		Male	1.00		1.00	
	Trust in regular source of care	Little/not at all	0.87 (0.56–1.34)	0.5262	0.72 (0.41–1.26)	0.2510
		Sufficiently	0.76 (0.61–0.96)	0.0214	0.67 (0.51–0.90)	0.0075
		Quite a lot	0.97 (0.81–1.16)	0.7029	0.91 (0.71–1.16)	0.4259
		Not reported	0.63 (0.48–0.82)	0.0006	0.75 (0.52–1.08)	0.1235
		Very much	1.00		1.00	
Enabling factors	District level Median OOP Cost to outpatient facilities (GEL)		0.98 (0.97–0.99)	0.0032	1.00 (0.98–1.01)	0.9583
	Household consumption tercile	High	1.65 (1.38–1.97)	<0.001	1.14 (0.9–1.45)	0.2758
		Medium	1.29 (1.07–1.54)	0.0064	1.09 (0.86–1.38)	0.4663
		Low	1.00		1.00	
	Median household age		1.01 (1.00–1.01)	0.0597	1.02 (1.01–1.02)	<0.001
	Physician density per 1000 population in the district		1.04 (0.97–1.11)	0.2701	0.95 (0.86–1.05)	0.3108
	Residence	Urban	1.12 (0.84–1.49)	0.4318	1.06 (0.75–1.49)	0.7509
		Rural	1.00		1.00	
	Travel time to regular source of care facility		1.00 (1.00–1.00)	0.7436	1.00 (0.99–1.00)	0.0255
	Type of closest facility	Hospital	1.17 (0.92–1.49)	0.1880	1.02 (0.75–1.39)	0.9089
		Village ambulatory clinics	1.42 (1.05–1.92)	0.0240	1.18 (0.81–1.73)	0.3837
		Others	1.12 (0.72–1.73)	0.6153	0.97 (0.5–1.87)	0.9322
		Polyclinic	1.00		1.00	
	Type of insurance	MIP	1.05 (0.87–1.26)	0.6108	1.21 (0.93–1.58)	0.1472
		Other insurance	1.1 (0.75–1.61)	0.6209	1.36 (0.81–2.27)	0.2494
		No insurance	1.00		1.00	
	Type of regular source of care facility	Hospital	0.84 (0.66–1.08)	0.1774	0.97 (0.67–1.41)	0.8618
		Others (incl. VAC)	0.94 (0.53–1.69)	0.8462	1.09 (0.6–1.98)	0.7827
		Polyclinic	1.17 (0.91–1.51)	0.2123	1.58 (1.06–2.37)	0.0254
		None	1.00		1.00	
Environment	Region	Tbilisi	1.15 (0.74–1.8)	0.5281	1.83 (0.99–3.4)	0.0542
		West	1.29 (1.07–1.54)	0.0061	1.30 (0.99–1.71)	0.0633
		East	1.00		1.00	
Confounder	Disease type	Chronic with exacerbation within 30 day prior to the survey	0.71 (0.54–0.93)	0.0122	0.82 (0.64–1.05)	0.1203
		Chronic illness only	0.03 (0.02–0.03)	<0.001	1.20 (0.93–1.55)	0.1517
		Acute illness only	1.00		1.00	
	Year	Year 2010	0.80 (0.67–0.97)	0.0205	1.27 (1.00–1.61)	0.0487
		Year 2007	1.00		1.00	

*CL* confidence limits, *GEL* Georgian Lari, *OOP* out-of-pocket

### Self-perceived health

When all other determining factors are held constant, self-perceived health, as a proxy for an individual’s health need, emerged as a strong predictor of outpatient service use. Consequently, those who perceived their health as ‘poor/very poor’ or ‘fair’ were significantly more likely to opt for outpatient care as opposed to no care or self-treatment.

### Predisposing individual factors

Age groups revealed different outpatient utilisation patterns. Children below 14 years compared to people aged 45–64 were 2.02 and 2.85 times more likely to choose outpatient care over not treating or self-treating, respectively. When faced with a health problem, individuals aged 15–44 were 1.52 times more likely to opt for outpatient care over self-treatment (*P* < 0.01). However, if a person was above 64 years old and all other factors were equal, no significant difference existed between comparison groups regarding outpatient utilisation.

The higher an individual’s educational attainment, the higher were the odds of using outpatient services. For example, people with a college or higher degree were 1.27 times more likely (*P* < 0.05) to choose outpatient care over no treatment compared to individuals with less than high school education. However, education had no influence when choosing between outpatient or self-treatment.

Concerning ethnicity, Armenians were 1.52 and 3.60 times more likely than Georgians to choose outpatient care over no treatment or self-treatment, respectively (*P* < 0.01), while other ethnic minorities, when compared with Georgians, were more likely to self-treat than to choose outpatient care (odd ratio (OR) = 0.63, *P* < 0.05).

Marital status did not seem to influence the choice of treatment significantly. However, sex had a significant influence; females were 15% more likely to use outpatient services compared to males (OR = 1.15, *P* < 0.05). Nonetheless, females and males did not seem to differ in behaviour when choosing outpatient care utilisation over self-treatment.

### Enabling factors

Median OOP payments for outpatient services in each district emerged as a significant barrier for outpatient service use. Namely, a one GEL increase in OOPs reduced the odds of seeking outpatient services by 2% (*P* < 0.01) versus no utilisation. OOPs did not impact an individual’s decision when choosing between outpatient care and self-treatment.

Furthermore, when all other factors are held constant, being in the top or medium wealth tercile increased the odds of choosing outpatient service over non-treatment by 65% and 29%, respectively (OR = 1.65, *P* < 0.01; OR = 1.29, *P* < 0.01). However, wealth is not a significant predictor when choosing between outpatient service and self-treatment.

A 1-year increase in median household age increased the odds of choosing outpatient care over self-treatment by 2% (OR = 1.02, *P* < 0.001). We also found that a 1-minute increase in travel time to a regular source of care decreased the odds of outpatient utilisation versus self-treatment by 1% (OR = 0.99, *P* < 0.05).

The urban-rural location of the household, the supply of physicians, the regular source of care establishment, and the type and availability of insurance had no influence on outpatient service use when adjusted for all other factors. The exceptions were polyclinics as the site for the regular source of care, which had a significant and positive influence on outpatient utilisation versus self-treatment (OR = 1.58, *P* < 0.05).

When comparing regions, only residents of Western Georgia had a higher odds of outpatient utilisation over no use (OR = 1.29, *P* < 0.01).

When all other factors were held constant, we noted changes in usage patterns between 2007 and 2010. Namely, the odds of outpatient utilisation versus no treatment declined (OR = 0.80, *P* < 0.05), i.e. in 2010, more people decided not to treat when ill; at the same time, people became 27% more likely to opt for outpatient care versus self-treatment (OR = 1.27, *P* < 0.05). Finally, our analysis revealed the significant and negative influence of chronic illness on outpatient service use. The odds of outpatient service utilisation for patients with chronic conditions was 97% less (OR = 0.03, *P* < 0.01) compared to those reporting acute health problems. Moreover, those reporting exacerbations of a chronic disease within 30 days prior to the survey were 29% less likely to seek outpatient care (OR = 0.71, *P* < 0.05) compared to those who only had acute illnesses.

### Study limitations

When interpreting these results, the data and methodological limitations should be taken into consideration. In this study, we aimed to measure the utilisation of chronic and acute patients. However, due to limitations imposed by the survey tool, we were not able to establish a sequence of outpatient visits for an individual, i.e. whether the first visit was made to a family physician on a primary care level, who subsequently referred an individual to a specialist. With the ability to measure sequence, we may have used a two-part regression model, which would have considered the influence of pre-exposure to a family physician on further health service use, thus making the findings more rigorous.

Secondly, when evaluating the need, we relied only on self-perceived health status. However, the available literature also differentiates between perceived and assessed needs and suggests using both [17]. Assessed need represents a professional judgment about people’s health status and their need for medical care. Consequently, experience with the disease and a past encounter with a provider enable individuals to differently think about their health status and take a different course of action when seeking service [17, 31]. While several authors [32, 33] documented a stronger relationship between assessed need and service use compared to the self-perceived measure, the limitations of the survey tool meant that we were unable to include evaluated need in our analysis. Consequently, the indicator of need used in our model might be an underestimation of real needs.

Thirdly, our measurement of ‘other private insurance’ does not account for the diversity of benefits covered by different insurance policies. Therefore, we treat the availability of private insurance as a dichotomous variable without further specification, which may minimise the actual value of insurance as an enabler in our model. However, this weakness is mitigated by the fact that (1) prevalence of private insurance is very low (≈4%) and (2) the government subsidised MIP is more prevalent (–22%) and uniform throughout the country, thus certainly measuring the actual enabling effect, if any, in our model. Furthermore, our paper is more focused on evaluating the enabling effect of public financing as opposed to private insurance.

Fourthly, our study did not capture the existence of multiple coexisting chronic diseases in individuals categorised as having a chronic illness, while the evidence suggests that such comorbidity may be associated with increased healthcare utilisation [34]. Thus, real usage patterns within the chronic cases may differ from those reported by our study.

Finally, in a cross-sectional household survey such as HUES, the relationships between variables can be performed with some degree of confidence but inferences of causation, if any, should be made with caution.

## Discussion

To operationalise the findings of our study, we assessed the determinants of patient behaviour through a mutability lens, i.e. which determinants could be modified using policy tools to bring about behavioural change. For example, demographic factors are judged as having low mutability, while beliefs could be assessed as having medium mutability. On the other hand, some enabling factors have higher mutability and could be relatively easily changed through appropriate policy actions [17].

Consequently, using this mutability lens, we first start off by discussing enabling factors, and then discuss the role of predisposing factors.

### Enabling factors

The results of the study show that physician density and urban-rural residence did not have any impact on utilisation rates. This phenomenon could be explained by the relatively adequate supply of inputs for service provision in Georgia; for example, the HUES 2010 found that 93.6% of the urban population and 77.8% of the rural population live within 30 minutes of a healthcare facility. Moreover, population reports indicate that the mean number of days that doctors are present at the village healthcare facility was equal to 4.92 days per week in 2010, while urban services have even better availability of physicians [14]. Consequently, at this point, unless the situation changes, interventions aimed at further expanding the network of providers to reduce travel time to a facility or improving the availability of doctors are not expected to affect utilisation rates significantly. On the other hand, the relationship between the level of OOPs, household wealth and service use certainly deserve greater attention. While findings show that OOPs and household wealth did not determine the choice between self-treatment and outpatient services use, both determinants were strong predictors for choosing outpatient care over no treatment. Consequently, public financing to expand the benefits package with the services needed by chronic patients (such as diagnostics and laboratory services, or outpatient prescription drugs for chronic conditions, etc.) and which are currently being paid by the patients has the potential to increase outpatient service use. Based on our results, every one GEL reduction in the costs to patients could increase the odds of outpatient service utilisation by 2%, when all other factors are held constant. The impact of such subsidies could be even greater if expanded benefits for chronic patients are primarily focused on the poorest, using the same targeting mechanisms that are operationalised under MIP. Implementing such approaches has the potential to deliver on equity objectives beyond utilisation growth. In addition to our findings, the Rand Health Insurance Study [35] suggests that reduced cost to patients at the point of care with the help of insurance increases utilisation rates when subsidies are well targeted at the factors that impede service use.

The arguments for benefit package expansion are further supported by the fact that having insurance, especially the state funded one (MIP), had no impact on the outpatient utilisation rates – a finding that was also documented by other studies [2, 16]. This finding is not surprising in a Georgian context because MIP benefits were mostly oriented towards inpatient services with limited outpatient coverage, especially those needed by the chronic patients [1]. Consequently, the benefits package failed to cover the bulk of essential outpatient services adequately and, most importantly, drug benefits for the chronically ill, which are one of the primary cost drivers for health services and a source of catastrophic health expenditure in Georgia [36]. The cost of drugs for chronic diseases remains the primary trigger for increased health spending and a potential source of catastrophic health spending even for MIP beneficiaries. Pharmaceutical spending accounts for up to 60% of household’s healthcare costs, while a chronic patient’s drug expenditure amounts to 86% of annualised recurrent expenditure [15].

The need to expand benefits afforded to chronic patients is further supported by confounders in our analysis. Specifically, the odds of choosing outpatient services versus no treatment was extremely low for chronic patients when all other factors were equal (OR = 0.03, *P* < 0.01). Moreover, there was no statistically significant difference in choosing outpatient services over self-treatment in this group. All of this further adds to the argument that more attention needs to be paid to the services and pharmaceuticals covered by the state-funded benefit package, which has the potential to facilitate utilisation by chronic patients, eventually improving their health [2] and delivering on equity objectives as well.

### Predisposing factors

After adjusting for need, education and sex emerged as predictors of outpatient service utilisation versus no treatment, but had no influence on using formal service provider versus self-treatment. As documented elsewhere [37], the educational attainment of an individual is a strong determinant of making a decision to treat or not [38, 39]. Consequently, well-educated patients were more prone to seek care. However, the mutability of this determinant is only possible through a long-term investment in the educational sector, which is beyond the influence of the health sector and is therefore not discussed here. Nevertheless, it deserves attention when developing a multi-sectoral, long-term strategy for health improvements.

Another predisposing factor for health service use was the age of a patient. Children aged between 0 and 14 were most likely to be treated by a healthcare provider, followed by the 15–44 age group, which chooses outpatient services versus self-treatment (OR = 1.52, *P* < 0.01). People aged over 64 also had higher odds of choosing outpatient services, but this was not statistically significant. Consequently, based on the data in Table 2, the lowest odds for service use versus self-treatment was found among those aged 45–64 years. The fact that those aged 45–64 years have the highest disease burden caused by cardiovascular diseases, neoplasms, diabetes, urogenital, blood, and endocrine diseases, which amount to 67% of the total disease burden for this age group [40], suggests either that the current healthcare system of Georgia fails to detect these conditions in a timely manner, and/or that the benefits included in the state-funded programs are inadequately tailored to the needs of this group to attract them to healthcare facilities. That said, it should be noted that similar age gaps in health utilisations are reported in other LMICs [8, 41, 42].

The next important determinant for outpatient use was sex. Overall, females were 15% more likely (OR = 1.15; *P* < 0.05) to seek outpatient services than men. These findings do not differ from the evidence available elsewhere [37]. However, based on the burden of disease data produced by the Institute for Health Metrics and Evaluation, 45- to 64-year-old males in Georgia have a 1.6-fold higher disease burden caused by NCDs compared to females since NCDs are 93% more prevalent among men in this age group [40]. Consequently, it is important to better address sex- and age-specific health needs through appropriate policy interventions, where possible. Policy responses could entail selecting the most prevalent diseases within the 45–64 age group and using national clinical guidelines to select and subsidise the diagnostics, drugs and other costs required for treating these conditions. This policy response could also be operationalised by re-designing the benefit package.

Finally, interesting findings also emerged regarding ethnicity as a determining factor for service use. Among Armenians, ethnicity emerged as a strong predictor for outpatient service use as well as for seeking treatment from formal providers. This population had higher odds (OR = 1.52) of using outpatient care versus no care (*P* < 0.01) and an OR = 3.6 of choosing outpatient care over self-treatment (*P* < 0.01) compared to Georgians. On the one hand, even though previous studies reported higher access barriers to family planning and antenatal services among Azeri and Armenian women [43], our findings reveal that ethnic Armenians, who comprise 4.31% of the total population [44], have better access to services than ethnic Georgians and are not marginalised by the system. Nevertheless, the lower OR for other minorities points to remaining problems in the system and highlights a need that should be addressed on a policy level. Factors negatively affecting other minorities could be numerous, such as language barriers [45], cultural beliefs and values, and require further exploration to arrive at possible policy solutions. However, such explorations are beyond the scope of this paper and should become the subject of other research.

## Conclusions

Previous evidence from Georgia showed that, after the healthcare financing reforms of 2007, chronic patients utilised fewer services compared to acute ones. In this paper, we have presented the determinants that impede service use. In particular, our findings indicate that low income, 45- to 64-year-old Georgian males with low educational attainment who suffer from chronic health problems have the lowest OR compared to the rest of the population for using outpatient services. Using a mutability lens for selecting possible policy responses, we think it would be important for Georgia to choose the most epidemiologically prevalent NCDs that place a significant disease burden on the 45–64 age group. Secondly, it would be necessary to expand the benefit package and subsidise those diagnostic and treatment services that are needed to care for these common conditions, including outpatient prescription drugs. Thirdly, in order not only to drive outpatient utilisation but also to deliver on equity objectives, the government may want to consider more expanded and comprehensive benefits for the poor, with no or very small co-payment. The operationalisation of these proposed approaches would require more evidence-based decision-making when re-designing publicly funded healthcare benefits as well as making them more people centred.

To conclude, Andersen’s behavioural model seems to be helpful in selecting and targeting determinants impeding outpatient service use and informing possible policy choices, especially those linked to benefit package design. The information that we have presented has the potential to inform decisions aimed at delivering better protection against ill health arising from NCDs in Georgia and other LMICs facing comparable challenges in access to and utilisation of healthcare services by individuals with chronic conditions.

## Abbreviations

GEL: Georgian Lari
HUES: health utilisation and expenditure survey
LMIC: low- and middle-income countries
MIP: medical insurance for the poor
NCDs: non-communicable diseases
OOP: out-of-pocket
OR: odds ratio

## Footnotes

1 Starting from 2013, the Government embarked on Universal Health Coverage Agenda and expanded state-subsidised benefits, comparable to those offered under MIP, to the whole population. To evaluate the results of this initiative, in 2014, a health utilisation and expenditure survey was repeated and WHO and the World Bank supported the Government with survey data analysis. Nevertheless, at the time of this paper submission (December 2016), neither the dataset nor the complete technical report of this study had been released into the public domain. Instead, the high-level results of the survey were communicated in a short presentation prepared by the World Bank and WHO, which is not publicly available. Consequently, our analysis only speaks to the evidence arising from the 2007 and 2010 HUES datasets and regretfully does not include the findings stemming from the 2014 survey.
